# The utility of peripheral stereopsis

**DOI:** 10.3389/fnins.2023.1217993

**Published:** 2023-09-19

**Authors:** Preeti Verghese

**Affiliations:** Smith-Kettlewell Eye Research Institute, San Francisco, CA, United States

**Keywords:** macular degeneration, amblyopia, strabismus, coarse stereopsis, periphery

## Abstract

This perspective article makes the case for evaluating and training peripheral stereopsis, particularly when the central visual field is compromised in one or both eyes. Examples of clinical conditions that preferentially affect the central visual field include macular degeneration, which affects the central macular region in one or both eyes, and amblyopia where the central field is often affected in one eye, but the peripheral field is largely intact. While binocular acuity may be preserved when the monocular central field of one eye is affected, fine stereopsis is compromised because it requires intact vision in corresponding locations in the two eyes. Even in these clinical conditions, recent studies that map stereoacuity at locations across the visual field demonstrate that the periphery supports coarse stereopsis, and that training efforts to use residual stereopsis may have greater benefit if they take this finding into account.

## 1. Introduction

When functional vision is impaired in the central part of the visual field where acuity is highest, it is often reported by the individual and confirmed by a simple acuity test. This is particularly true if the vision loss occurs in both eyes. However when vision loss occurs in only one eye or in non-overlapping parts of the two eyes, visual fields are largely intact and the vision loss may go unnoticed. In these cases, stereopsis is impacted in the regions corresponding to vision loss in either eye. This perspective article discusses two clinical conditions (macular degeneration and amblyopia) that are associated with a loss of stereopsis in the central visual field, and makes the case that residual stereopsis is mediated by the periphery.

## 2. Evidence for peripheral stereopsis

### 2.1. Peripheral stereopsis in macular degeneration

**Figure 1:**
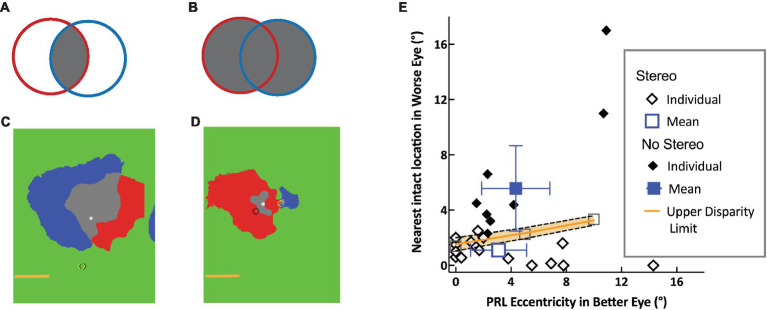
Schematic of Overlapping left (red) and right (blue) scotomas, aligned on the fovea (asterisk): schematic and data. **(A)** The intersection of the scotomata (gray), forms the binocular scotoma. **(B)** The union of the two eyes’ scotoma forms the more extensive stereo-blind zone. **(C,D)** Scotoma of two individuals showing significant and little overlap respectively, with the two eyes’ scotomata aligned on the fovea (white asterisk). The left and right eye PRLs are shown by the yellow and black circles, respectively. The horizontal line indicates 5°. **(E)** Separation between healthy locations in the two eyes. The distance between the PRL in the better eye and the nearest intact location in the other eye is plotted as a function of PRL eccentricity. Open and filled diamonds show data for individuals with and without measurable stereopsis, respectively, along with mean data (blue) and 95% confidence limits. The upper disparity limit (orange) represents the upper bound of retinal separations that support stereopsis [from Verghese and Ghahghaei (2020)].

I will first consider age-related macular degeneration (AMD), a condition where the region of functional vision loss is directly related to the area of the retina that is affected. AMD has a high prevalence, affecting about 13% of the U.S. population over age 40 (Rein et al., 2002). The disease occurs in two forms (dry and wet) and preferentially affects the macula, which spans the central 16° (Schuchard et al., 1999; Cheung and Legge, 2005; Hood, 2015). The type of functional loss depends on how much the scotomata in the two eyes overlap. When they occur in overlapping locations in the two eyes that includes the fovea (Figure 1A), the resultant binocular central field loss (CFL) can significantly impact daily life, particularly tasks that require high-acuity vision, such as reading, recognizing faces, and finding items of interest (Legge et al., 1992; Fine and Peli, 1995; Fletcher et al., 1999; Chung, 2011). Moreover, even when the scotomata in the two eyes overlap only partially (Figure 1B) or not at all, individuals experience loss of stereopsis in parts of the visual field that correspond to a scotoma in *either* eye. In these instances, the areas with loss of stereopsis are typically more extensive than the binocular scotoma. In the extreme case when there is a scotoma in only one eye, binocular visual fields may be intact, but stereopsis is impacted. Thus, while individuals become acutely aware of their vision loss when they have a binocular scotoma, non-overlapping scotomata can impact tasks that benefit from stereopsis such as eye-hand coordination (Melmouth et al., 2009; Cao and Markowitz, 2014; Verghese et al., 2016), walking (Hayhoe et al., 2009; Buckley et al., 2010; Bonnen et al., 2021), and negotiating steps and curbs.

Curiously, there are only a few published studies that report the incidence of stereopsis in individuals with AMD. One of these, Cao and Markowitz (2014) reported that only 11 out of 27 participants with AMD had stereopsis better than 340 arc sec using the Frisby Stereotest (Stereotest Ltd., Sheffield United Kingdom), while the other study (Verghese et al., 2016) reported that only 6 out of 15 participants with AMD had stereopsis better than 1,000 arc sec. These numbers are consistent with a longitudinal study of visual function in elderly participants (Haegerstrom-Portnoy et al., 1999). A follow-up to this study (unpublished) measured stereopsis among individuals with AMD (*n* = 42) and age-matched controls (*n* = 212). Only 38% of the participants with AMD had stereopsis of 340 arc secs or better on the Frisby Stereo test, compared to 80% of the age-matched controls. These data along with the results of the other two published studies (Cao and Markowitz, 2014; Verghese et al., 2016), show that only about 40% of individuals with AMD have measurable stereopsis in the range 340 to 1,000 arc sec.

However, if the criterion for residual stereopsis is relaxed to 2000 arc sec (the upper wings of the Randot Butterfly), then 16 out of 25 participants with AMD (64%) have measurable stereopsis (Verghese and Ghahghaei, 2020). As the central retina is affected in AMD and the periphery is relatively intact, it is likely that it is the periphery that is mediating this coarse stereopsis. We have specifically examined the ability of the periphery to mediate stereopsis in AMD using three approaches.

The first is a hand-eye coordination task that demonstrated the practical benefit of residual stereopsis (Verghese et al., 2016). In this task, individuals with AMD who had residual stereopsis in the periphery were better able to do the task under binocular compared to monocular viewing, with a binocular benefit that was proportional to residual stereo sensitivity (1/stereoacuity).

The second approach is a direct test of the hypothesis that depth from disparity is compromised in any part of the visual field corresponding to vision loss in either eye. By using a novel method to map stereopsis across the visual field with a local disparity target, we were able to compare the resulting stereoperimetry map with the prediction that the stereoblind region would be determined by the union of the scotomatous regions in the two eyes (Verghese et al., 2016). Our results are consistent with this hypothesis, for a range of scotoma sizes in the two eyes from symmetric overlapping scotomata in the two eyes to asymmetric scotomata, including monocular field loss (see Figures 1C,D for examples). Furthermore, we were able to show that all participants had coarse stereopsis outside the predicted stereoblind zone. Clearly, peripheral regions with intact retina outside the scotomata in both eyes are able to mediate depth from disparity. However, individuals with asymmetric scotomata in the two eyes are not always aware that their peripheral retina can mediate useful stereopsis. This is because central field loss causes them to adopt a preferred retinal locus (PRL), often based on a compromise between good acuity and a desire to get the scotoma out of the lower visual field, which is important for most-eye-hand coordination tasks. As acuity declines sharply with eccentricity, individuals tend to pick a location as close to the fovea as possible. In cases of asymmetric vision loss, the PRL location in the better eye is closer to the fovea even though the retina in the worse eye is non-functional at the corresponding location (Tarita-Nistor and Mandelcorn, 2022). This means that the PRL location is being optimized for acuity at the cost of functional stereopsis.

The third approach tests the hypothesis that residual stereopsis is determined by the distance between intact retinal locations in the two eyes (Verghese and Ghahghaei, 2020). The largest separation between corresponding features that mediates the perception of depth from disparity at a given eccentricity is the upper disparity limit. This upper disparity limit (orange line in Figure 1E) increases with eccentricity (Ghahghaei et al., 2016) and is directly related to the size of the largest receptive fields that are sensitive to disparity, at that eccentricity. Therefore, receptive field size at a given eccentricity determines the maximum separation of corresponding features in the two eyes that can be coded by the same disparity-sensitive unit. We measured the distance between the PRL in the better eye and the nearest location of healthy retina in the worse eye and showed that individuals with AMD have coarse stereopsis (open diamonds in Figure 1E) when the separation between these retinal locations is smaller than the upper disparity limit measured behaviorally. Because the disparity limit is about a factor of 2 to 4 times larger at an eccentricity of 10° compared to its size at the fovea (Ghahghaei et al., 2016), individuals with asymmetric scotomata in the two eyes are less likely to have measurable stereopsis when they have a PRL close to the fovea, compared to when the PRL is at a more eccentric location. Those without measurable stereopsis (filled symbols in Figure 1E) have a separation between their better-eye PRL and intact retina in the other eye that is larger than the disparity limit at the eccentricity of the PRL. As PRLs are known to adapt to the needs of a task (e.g., Sullivan et al., 2008; see also Lei and Schuchard, 1997, and Duret et al., 1999), it is an open question whether these individuals can be made more aware of their potential for peripheral stereopsis and whether they can switch to a more eccentric PRL for tasks that benefit from stereopsis such as eye-hand coordination and navigation.

### 2.2. Peripheral stereopsis in anisometropia and small-angle strabismus

Amblyopia, which is also associated with a loss of stereopsis, is fundamentally a developmental abnormality of visual cortex, rather than the retina. It affects 3 to 5% of the population worldwide (Holmes and Clarke, 2006) and is often associated with a failure of the two eyes to work together effectively (McKee et al., 2003). Amblyopia is commonly caused by misalignment of the eyes (strabismus), chronic optical blur due to unequal refractive error in the two eyes (anisometropia), or a mixture of strabismus and anisometropia during early childhood. Nevertheless, a significant proportion of amblyopes and strabismics have coarse stereopsis [50% of anisometropic amblyopes and up to 40% of strabismics depending on the age of corrective surgery, see (Birch and Wang, 2009; Levi et al., 2011)]. When present, stereoacuity in amblyopia and strabismus is often worse than 200 arc sec (Parks, 1969), a value associated with eccentricities of 6° or more in normal participants (Siderov and Harwerth, 1995; Wardle et al., 2012; Ghahghaei et al., 2016). Thus, the issue may be a lack of functional stereopsis at the point of gaze (fovea of fellow eye) when the other eye is strabismic and/or amblyopic, potentially impacting grasp, balance, and general visual motor coordination (Servos et al., 1992; Hrisos et al., 2006; Melmouth et al., 2009; Niechwiej-Szwedo et al., 2019; Kelly et al., 2020). Yet, the individual may perceive depth in 3D movies (e.g., Bridgeman, 2014). One explanation for the ability of 3D movies to evoke compelling percepts of depth in individuals with poor foveal stereopsis is that unlike standard tests for stereoacuity, the large screen stimulates the periphery where there is residual capacity for stereopsis (see caveat regarding dynamic stereopsis below). To test the hypothesis that stereopsis in anisometropia and small-angle strabismus may be mediated by the periphery, we have embarked on a series of studies to measure local stereopsis across the visual field.

**Figure 2:**
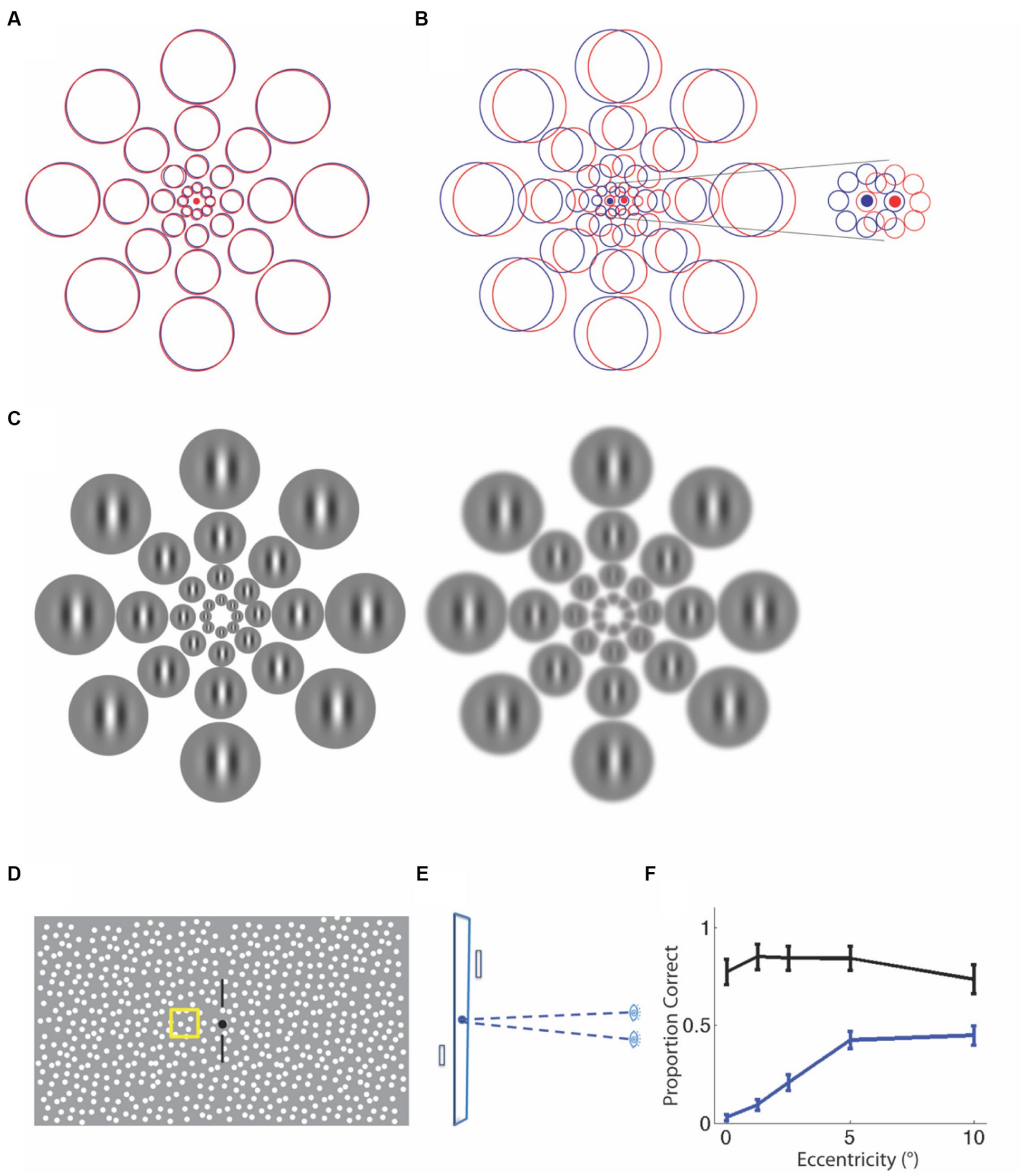
Explanations for a central stereo deficit in strabismus and anisometropia. **(A)** Normal alignment of receptive fields in the two eyes with overlapping left-(red) and right- eye(blue) receptive fields. **(B)** An example of a misalignment of the eyes due to strabismus. The receptive fields do not overlap, particularly at the smallest scales at the center (see inset). The same misalignment leads to partial overlap at peripheral locations, providing a substrate for stereopsis. **(C)** An example of anisometropia with optical blur in one eye, resulting in the loss of fine detail and feature contrast. The receptive field locations are now shown as Gabor patches that scale with eccentricity. It is clear that the same blur obliterates spatial modulation information at fine scales in the center, but leaves the coarser scale information in the periphery fairly intact, allowing for the peripheral matching of corresponding features. Schematic for stereoperimetry stimulus. **(D)** Frontal View. **(E)** Plan View. On every trial, a single patch was presented in front of or behind the fixation plane and observers judged the sign of depth. **(F)** Proportion of trials seen and identified correctly for sign of depth change. Anisometropic observers (blue) are significantly worse than controls (black), particularly at the fovea.

Here is the rationale for why stereopsis might be impacted in the center of the visual field. Let us consider the case of normal visual development where the two eyes are roughly aligned and have similar refractive error. Figure 2A illustrates the increase in receptive field size with eccentricity at the level of visual cortex (Rovamo and Virsu, 1979) for each eye (red, left eye; blue, right eye). In a typical individual, these receptive field locations overlap, so that cortical neurons receive input from corresponding parts of the visual field in the two eyes. In strabismus, because of a misalignment of the two eyes, the overlap of the two eyes’ representations depends on the angle of deviation between the eyes. For small angles of deviation, there is no overlap of the inputs from the small receptive fields at the center of the visual field (see Figure 2B and inset), but there is partial overlap of the inputs at larger eccentricities in the periphery because of the larger receptive field size. Thus, neurons in the periphery are able to get input from both eyes, a prerequisite for stereopsis. As the deviation between the eyes increases, the size of the central stereoblind zone increases.

There is some evidence for residual stereopsis in the periphery of individuals with small angles of strabismus. One previous study (Kitaoji and Toyama, 1987) measured the ability of individuals with strabismus to detect large static disparities (0.5, 1 and 2°) in the fovea and showed that only individuals with deviations smaller than 2 degrees (3.5 prism diopters) had coarse foveal stereopsis. When these disparities were shown in the periphery at 10 and 20° eccentricity, individuals with slightly larger deviations of up to 5° (8.8 prism diopters) were able to detect depth from disparity. This and other studies (Sireteanu et al., 1981; Sireteanu, 1982) have also found that when the disparity of the target changed dynamically rather than remaining static, individuals with even larger deviations [up to 16° as reported by Kitaoji and Toyama (1987)] were able to detect these changes in the periphery. As a dynamic-disparity stimulus (generated by moving corresponding features in opposite directions in the two eyes) evokes responses in mechanisms sensitive to changing disparity as well as those that are sensitive to interocular velocity difference (Roker et al., 2009; Nefs et al., 2010) it is not clear that the reports of dynamic stereopsis in the periphery of strabismics indicates a true sensitivity to disparity. We have replicated Kitaoji & Toyama’s result by demonstrating peripheral sensitivity to disparity-defined targets embedded in a static random-dot stereogram (see Figure 2D for the display). Three individuals with strabismus who had angles of deviation <5°, were not able to detect the 20 arc minute disparity target in the central visual field, but were able to detect it at the largest eccentricities tested.

Now consider the case of anisometropia, where uncorrected refractive error in one eye effectively blurs the fine features to which the central retina is usually selective, and reduces their contrast. Figure 2C is a schematic of the observed pattern of increasing receptive field size with eccentricity with a superimposed Gabor stimulus that also scales with eccentricity. It is clear that blur removes the fine details of the stimulus at smaller eccentricities and effectively reduces stimulus contrast at fine scales, providing a lack of features to match with the other eye’s input. This explains why the central visual field in an anisometropic amblyope might be stereo blind, consistent with reports of the loss of stereopsis at high frequencies (fine scales) with monocular blur (Li et al., 2016), as well as the detrimental effect of interocular contrast difference on stereoacuity (Legge and Gu, 1989).

To determine the pattern of stereopsis across the central 20° of the visual field, we used a method analogous to Humphrey perimetry, where we used local stereo targets that we scaled to eccentricity. Observers viewed a dichoptic random-dot display, maintained gaze at a fixation point while keeping nonius lines aligned, and judged whether a target was presented in front of or behind the fixation plane (Figures 2D,E), while fixation was monitored. The two eyes’ displays were aligned to compensate for deviation, if present. The target could appear on the cardinal or diagonal axes, at eccentricities of 0, 1.25, 2.5, 5, and 10° from fixation, at inter-trial intervals chosen randomly between 3 and 5 s. When the 20 arc minute disparity target was detected, observers reported the sign of depth; when there was no response to the target, it was counted as a miss. Figure 2F plots the proportion of target presentations where the target was detected *and* its sign of depth was judged correctly, averaged by eccentricity (unpublished data). The black symbols plot average data for 8 controls, whereas the blue data are for 4 anisometropic amblyopes. Compared to controls, anisometropic amblyopes are stereoblind at the fovea. Their ability to detect the disparity-defined target and judge the sign of depth improves with increasing eccentricity, indicating that peripheral stereopsis is present in anisometropic amblyopia.

## Conclusion

Taken together our studies indicate that in both macular degeneration and strabismus/ amblyopia, there is residual capacity for stereopsis in the periphery. This is relevant not only for understanding the crucial role of the periphery in stereopsis in these clinical conditions, but it also points to a need to revise our current approaches to both measurement and rehabilitation of stereopsis. Thus, stereo tests need to examine stereopsis across the visual field, not just at fixation. Furthermore, the dichoptic perceptual-learning paradigms to improve binocularity and stereopsis (Hess et al., 2010; Ding and Levi, 2011; Kelly et al., 2018) should specifically target peripheral locations.

## Author contributions

The author confirms being the sole contributor of this work and has approved it for publication.
